# Effect of Isomorphous Substitution on the Thermal Decomposition Mechanism of Hydrotalcites

**DOI:** 10.3390/ma7107048

**Published:** 2014-10-17

**Authors:** Sergio Crosby, Doanh Tran, David Cocke, El-Shazly M. Duraia, Gary W. Beall

**Affiliations:** 1 Department of Chemistry and Biochemistry, Texas State University, 601 University Drive, San Marcos, TX 78666, USA; E-Mails: sdcrosby@utexas.edu (S.C.); ed24@txstate.edu (E.-S.M.D.); 2 Department of Chemical Engineering, Lamar University, 4400 South MLK, Beaumont, TX 77705, USA; E-Mails: doanhtran@gmail.com (D.T.); deltagco@hotmail.com (D.C.); 3 Physics Department, Faculty of Science, Suez Canal University, Ismailia 41522, Egypt; 4 Physics Department, Faculty of Science, King Abdulaziz University, Jeddah, 21589, Saudi Arabia

**Keywords:** hydrotalcite, thermal decomposition, dehydroxylation, charge density, isomorphous substitution

## Abstract

Hydrotalcites have many important applications in catalysis, wastewater treatment, gene delivery and polymer stabilization, all depending on preparation history and treatment scenarios. In catalysis and polymer stabilization, thermal decomposition is of great importance. Hydrotalcites form easily with atmospheric carbon dioxide and often interfere with the study of other anion containing systems, particularly if formed at room temperature. The dehydroxylation and decomposition of carbonate occurs simultaneously, making it difficult to distinguish the dehydroxylation mechanisms directly. To date, the majority of work on understanding the decomposition mechanism has utilized hydrotalcite precipitated at room temperature. In this study, evolved gas analysis combined with thermal analysis has been used to show that CO_2_ contamination is problematic in materials being formed at RT that are poorly crystalline. This has led to some dispute as to the nature of the dehydroxylation mechanism. In this paper, data for the thermal decomposition of the chloride form of hydrotalcite are reported. In addition, carbonate-free hydrotalcites have been synthesized with different charge densities and at different growth temperatures. This combination of parameters has allowed a better understanding of the mechanism of dehydroxylation and the role that isomorphous substitution plays in these mechanisms to be delineated. In addition, the effect of anion type on thermal stability is also reported. A stepwise dehydroxylation model is proposed that is mediated by the level of aluminum substitution.

## 1. Introduction

Hydrotalcite (HT) is a layered double hydroxide, LDH. LDHs are an interesting class of anionic layered minerals that can readily be intercalated with a large variety of anionic materials, including large biomolecules, such as DNA. There are a great number of applications for LDHs, including as catalysts, as wastewater treatment additives, as gene delivery vectors and as a polymer additive to promote stability, such as in the processing of polyvinyl chloride [1,2,3,4,5]. HTs are based upon the brucite structure and have the general formula of Mg_1−X_Al_X_(OH)_2_A_X_^−^-yH_2_O, where A can be any anion, but is most commonly carbonate [6,7]. The structures of these materials consist of positively-charged, brucite-like hydroxide layers with negatively-charged interlayers. The layer spacings depend on the nature of the interlayer anions and the state of hydration, with the magnitude of the electrostatic attraction between layers and interlayers playing a significant role. In LDHs, the Mg can be substituted by almost any divalent metal cation, and aluminum can be substituted by any trivalent metal cation. The most common commercially available hydrotalcite is provided in the carbonate form and has an x-value of approximately 0.33. The thermal stability and mode of dehydroxylation is of particular interest in catalysis, since the LDHs are normally the precursors to the final mixed oxide catalysts. In the case of polymer stabilization, this thermal stability is of particular interest, since most thermoplastic polymers are processed in the range of 200 to 300 °C. 

In most past studies on the thermal decomposition of HTs, a complicating factor has been the simultaneous dehydroxylation and anion decomposition of the interlayers [8,9,10,11,12]. This results in overlapping of decomposition curves from thermal analysis and makes it difficult to determine the contributions due to dehydroxylation and anion decomposition. This is particularly acute in the carbonate form of HTs. The situation is further exacerbated by the fact that most of the studies have utilized HTs that have been prepared at room temperature. These precipitates are poorly crystalline with a very small particle size and high surface areas that promote the absorption of CO_2_ from the air as the carbonate ion. The propensity to convert to the carbonate form is a complicating factor that is overlooked in many cases. In order to circumvent this problem, the thermal decomposition of hydrotalcites containing chromate and molybdate anions were studied [13]. In these studies, the losses observed in the thermograms were divided into two broad classes. The first is the loss of physically absorbed water and waters of hydration below 240 °C. The second is the dehydroxylation above 240 °C, where two major steps occur. It is postulated that these two steps can be explained by the formation of intermediate oxyhydroxide species involving the aluminum cation. In contrast, a study of the chloride form of LDHs concluded that this two-step dehydroxylation is due to two types of hydroxyl groups. The hydroxides near or on the edges of the gallery leave first, followed by the hydroxyls in the interior of the gallery [14].

Therefore, in this study, HTs were synthesized hydrothermally to minimize any edge effects and to lessen the potential for carbonate exchange. These hydrotalcites were synthesized with x varying from 0.06 up to 0.3 to determine the role of aluminum in the dehydroxylation. The samples were analyzed utilizing X-ray diffraction, thermogravimetric analysis and mass spectrometry to identify the evolved gases (EGA) analysis. A stepwise dehydroxylation mechanism is proposed that supports a decomposition mechanism similar to that reported in the chromate and molybdate forms of hydrotalcites [13]. The proposed mechanism is strongly supported by the level of aluminum substitution in the lattice and similar results for the decomposition of Gibbsite, where the formation of intermediate aluminum oxyhydroxides are reported [15].

## 2. Results and Discussion

In most commercially available hydrotalcites and materials reported in the literature, the compounds tend to be of a formula [M^II^_1−x_M^III^_x_ * (OH)_2_]A^n−^, where x is close to 0.3 and the anion A is carbonate. Hydrotalcites have a strong tendency to convert to the carbonate form if exposed to air and humidity. This is particularly true of the room temperature precipitates utilized in the vast majority of studies reported in the literature. Once the carbonate form of hydrotalcite is established, it is difficult to exchange other anions into the gallery. Therefore, in this study, we synthesized the HTs in their chloride form to facilitate subsequent ion exchange reactions and to remove the effect of carbonate in thermograms. The ability to exfoliate plate-like materials is also affected by the charge density, and therefore, the hydrotalcites in this study have been further synthesized with nominal charge densities of 100, 200, 300, 400 and 500 meq/100 g. These samples have been studied by X-ray diffraction and various thermal decomposition techniques. The samples were synthesized under hydrothermal conditions to potentially lessen any edge effects as postulated in previous work [14] and to reduce the tendency for carbonate to exchange into the gallery. The hydrotalcite were synthesized at room temperature, 80, 130 and 150 °C. The carbonate contamination observed in this study for room temperature prepared material dictated that the research be conducted on the most crystalline material prepared at 150 °C.

### 2.1. X-ray Diffraction

**Figure 1:**
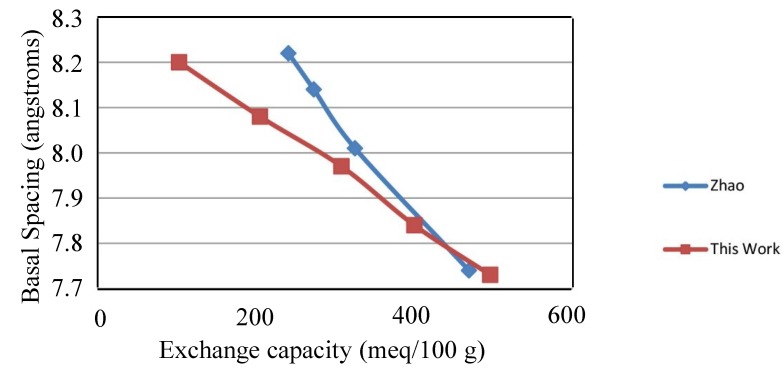
Basal d-spacing for hydrotalcites as a function of charge density in this study compared to Zhao [5].

Figure 1 gives the basal d-spacing for the series of hydrotalcites as a function of exchange capacity (charge density). The X-ray diffraction patterns for the series of chloride hydrotalcites of varying charge densities yield basal d-spacings that are very regular. The d-spacings are 8.19, 8.08, 7.97, 7.84 and 7.73 angstroms for 100, 200, 300, 400 and 500 meq/100 g, respectively. The results are also in reasonably good agreement with Zhao *et al*. [5]. The X-ray diffraction patterns for these materials have been published previously and are not included here [16]. The two series do diverge at lower charge density, which has been reported previously by Yun *et al*. [17], where the precipitation of HTs yielded different basal spacings depending upon the precipitation being carried out at constant or variable pH. This work precipitated the HTs at variable pH, while Zhao *et al*. [5], conducted the precipitation at constant pH. The contraction of the interlayer is a strong indication of how the increased charge holds the layers together more strongly. For a given anionic form of HT, the basal d-spacing is a fairly sensitive way to measure charge density. The a-axis for HTs are essentially constant and not affected by changes in charge density and anion substitution.

### 2.2. Thermal Decomposition

**Figure 2:**
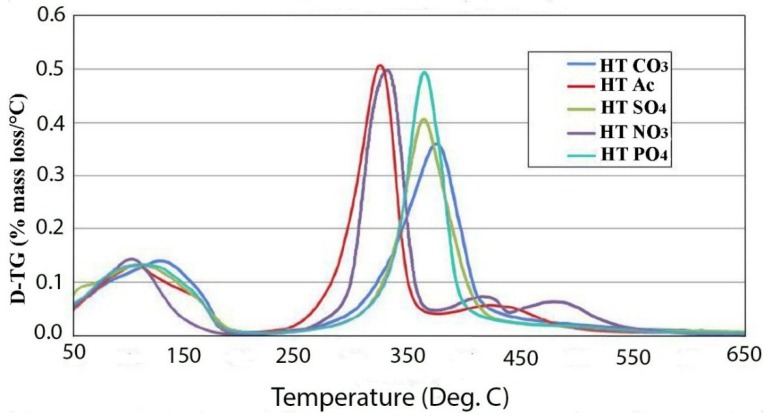
DTGA of various anionic forms of hydrotalcite.

The thermal decomposition of various anionic forms has been studied. The data does not include the originally synthesized chloride form, which will be discussed in detail later, but does contain data on the nitrate, acetate, sulfate, phosphate and carbonate forms. Figure 2 gives an overlay of the differential thermogravimetric analysis (DTGA) for all of the hydrotalcite forms. The first thing that stands out about these decomposition curves is that the monovalent anionic forms are significantly less thermally stable than the multivalent forms by approximately 50 °C. The chloride form has a decomposition peak at 320 °C; although it has a more complex decomposition curve, it is in line with this trend. This correlates well with the relative ease of exchange of the monovalent anion forms. Secondly, sulfate and phosphate anion-treated LDHs dehydroxylate at about the same temperature with a peak at about 370 °C. In contrast, the carbonate form has a thermal peak decomposition about a 10 to 15 degrees higher than other divalently charged anions. One reasonable explanation for this is that sulfate and phosphate are both tetrahedral molecules, while carbonate is trigonal planar. The flat configuration of carbonate allows it to lie parallel to the surface of the plates and interact better coulombically with adjacent plates. The dehydroxylation temperatures observed in this study for nitrate and carbonate are in disagreement with those found by Kloprogge *et al*. [12], where it was reported that in carbonate, the dehydroxylation occurred at 335 °C and nitrate and chloride at 412 °C and 370 °C, respectively. Vagvolgyi *et al*. [10] also reported much lower temperatures of dehydroxylation than observed in this study. The main differences that exist between this study and those cited are that the growth conditions were radically different. In our case, the data reported was on samples hydrothermally grown at 150 °C for 24 h, while the cited studies were precipitated at room temperature with little aging to allow crystallization. It has been shown that the crystallinity of hydrotalcites are a strong function of temperature and time of aging [5]. For example in that study, at 65 °C, it required 30 h to reach 85% crystallinity, and at 110 °C, it only took 3 h to reach 100% crystallinity. The crystallinity in that study was judged by peak width at half maximum in the X-ray diffraction patterns. This could indicate that the materials studied in [10,12] are not full hydrotalcites and may contain less stable amorphous metal hydroxyl complexes. This work is in good agreement with the thermal decomposition data by Rey *et al*. [11].

**Figure 3:**
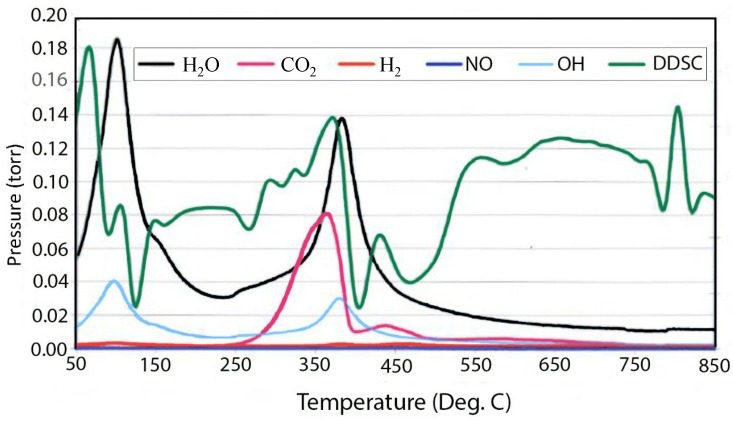
Double differential scanning calorimetry (DDSC) and corresponding evolved gas analysis for thermal decomposition of 400 meq/100 g hydrotalcite grown at 25 °C.

**Figure 4:**
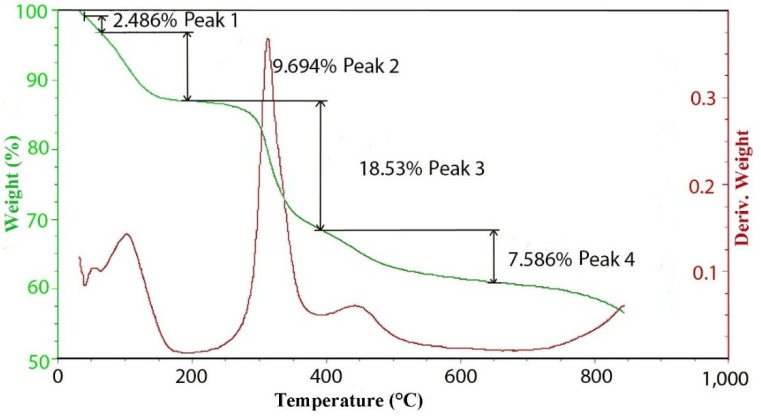
TGA and DTGA curves for the thermal decomposition of hydrotalcite with 100 meq/100 g grown at 150 °C.

The tendency of the room temperature precipitated material to absorb CO_2_ from the air can be seen in Figure 3, which contains the evolved gas data from the decomposition of 400 meq/100 g material precipitated at room temperature, which is supposed to be in the chloride form. It can be seen that there is a very large peak for carbonate decomposition and the release of CO_2_ in the mass spectrum. This is in contrast to the very small peak for carbonate in the material grown at 150 °C given in Figure 4. The thermal decomposition of the chloride form of hydrotalcite yields interesting results that are more complex and strongly dependent upon charge density or the degree of isomorphous substitution of aluminum. 

**Figure 5:**
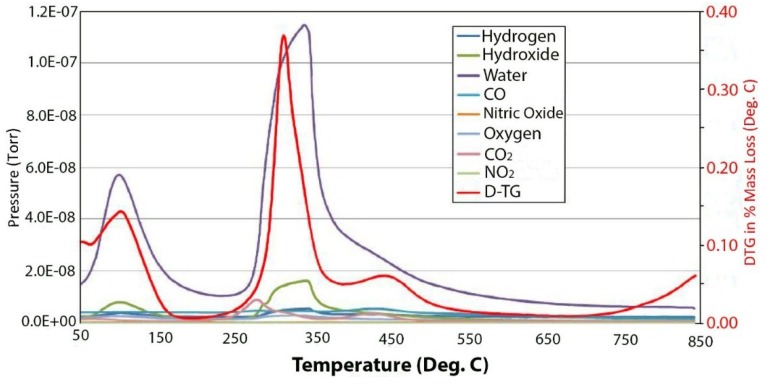
Mass spectrum of the off-gases from the thermal decomposition of 100 meq/100 g hydrotalcite.

Figure 5 contains the TGA and DTGA curves for the 100 meq/100 g sample of hydrotalcite. The weight loss curve can be divided into three main parts. The first is the loss of waters of hydration that occurs below 200 °C followed by the loss of hydroxyls at between 200 °C and 600 °C and, then, finally, a small additional weight loss above 600 °C. As mentioned previously, the peak of dehydroxylation occurs at about 320 °C. Calculating the theoretical weight loss for [Mg^2+^_.94_Al^3+^_.06_(OH)_2_]Cl^−^_.06_ gives a value of 29.71% weight loss upon dehydroxylation, while the observed loss corrected for waters of hydration yields 29.74%, which is in very good agreement. Analysis of the evolved gases coming from the thermal decomposition can be seen in Figure 4. The only significant gases coming from the thermal decomposition are water and its hydroxyl fragment that is fragmented in the ion source of the mass spectrometer. It should be noted that there is only trace amounts of CO_2_ from carbonate in the evolved gas. It is interesting to note that in pure brucite [14], the dehydroxylation occurs at between 267 °C and 331 °C. The next interesting observation is the changes that are observed in the thermal decomposition curves as a function of charge density or isomorphous substitution of aluminum. 

**Figure 6:**
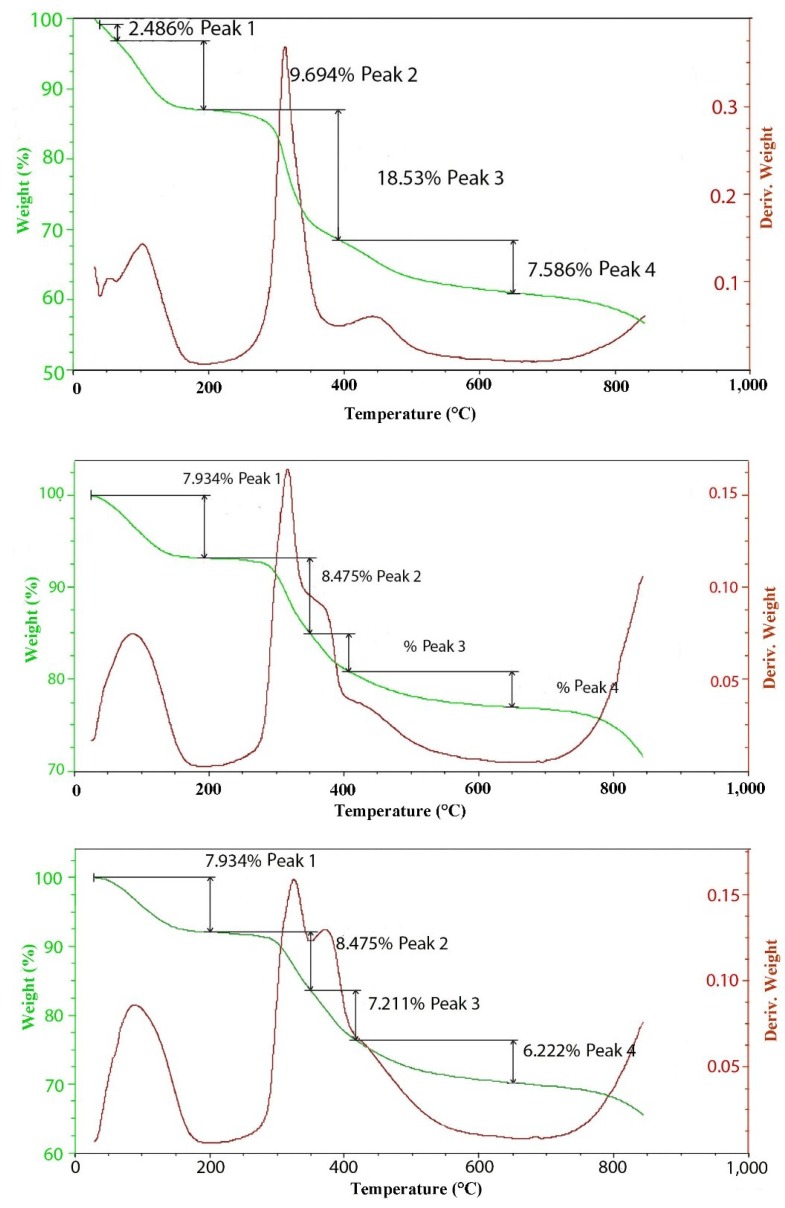
TGAs of 100 (top), 300 (middle) and 500 (bottom) meq/100 g hydrotalcites.

The thermal decomposition curves for 100, 300 and 500 meq/100 g hydrotalcites grown at 150 °C are included in Figure 6. It is interesting to note the changes in the shape of the differential TGA in the region where dehydroxylation is occurring. At the lowest charge density of 100 meq/100 g, the formula is [Mg^2+^_.94_Al^3+^_.06_(OH)_2_]Cl^−^_.06_, so the level of aluminum substitution is rather low. The peak of decomposition is centered at 320 °C, but is just slightly asymmetric with a hint of a shoulder on the upper temperature side. A second smaller peak occurs at around 450 °C. At 300 meq/100 g, the formula is now [Mg^2+^_.82_Al^3+^_.18_(OH)_2_]Cl^−^_.18_; the peak at 320 is still clear, but the shoulder is very pronounced and centered at 380 to 400 °C. Finally, at 500 meq/100 g, where the formula is [Mg^2+^_.71_Al^3+^_.29_(OH)_2_]Cl^−^_.29_, the shoulder is now emerging as a separate peak. This would indicate that the aluminum substitution quite profoundly changes the dehydroxylation mechanism. Unfortunately, the dehydroxylation peaks overlap to such an extent that assigning exact values to each step is imprecise. For this higher charge case, the theoretical loss of one water molecule per formula weight yields a loss of 25.98% with an observed value of 23.8%. Work done by Digne *et al*. [15] may provide a good explanation for the effect of aluminum substitution on dehydroxylation. In that work, the dehydration of aluminum hydroxide was studied. Starting with gibbsite (Al(OH)_3_), the initial dehydroxylation begins at around 250 °C. This first step leads to an intermediate phase of boehmite (AlOOH) that subsequently dehydroxylates to Al_2_O_3_ at 450 °C. If we take an idealized formula of [Mg^2+^_.667_Al^3+^_.334_(OH)_2_]Cl^−^_.334_ and make the formula whole numbers, we get [Mg^2+^_2_Al^3+^(OH)_6_]Cl^−^, which then can be dehydroxylated in steps. In the first step, one water molecule will be lost, giving [Mg^2+^_2_Al^3+^O(OH)_4_]Cl^−^, which yields of about 8.45% weight loss. The second step again is the loss of a second water molecule to go to [Mg^2+^_2_Al^3+^O_2_(OH)_2_]Cl^−^, which will result in an accumulative weight loss of 16.9%. The last step is the loss of a third water ending with a formula of [Mg^2+^_2_Al^3+^O_3_]Cl^−^, which would yield a total weight loss of 22.5%, which is in reasonable agreement with what was observed for the 500 meq/100 g of 23.8%.

## 3. Experimental Section 

### 3.1. Hydrotalcite Preparation

The initial precipitation of hydrotalcite was done by mixing the appropriate ratio of MgCl_2_·6H_2_O and AlCl_3_, to result in the desired anion exchange capacity, as illustrated in Table 1, in one liter of water with subsequent addition of NaOH to bring the pH to a value of 10. All solutions were purged with nitrogen to eliminate carbon dioxide, including NaOH solutions. The mixtures were then placed in a Parr reactor and heated to 80, 130 or 150 degrees Celsius for 24 h. The samples were then filtered and rinsed in Buchner funnels. The final anion exchange capacities were 103, 205, 308, 393 and 496 with details of each composition given in the work by Sanderson *et al.* [16].

**Table 1:** Synthesized compositions for target exchange capacity.

Exchange capacity meq/100 g	Formula Mg = 2^+^, Al = 3^+^, Cl = 1^−^	X Mg_(1−X)_Al_X_(OH)_2_Cl^−^_X_
100	[Mg_.94_Al_.06_(OH)_2_]Cl._06_	0.06
200	[Mg_.88_Al_.12_(OH)_2_]Cl._12_	0.12
300	[Mg_.82_Al_.18_(OH)_2_]Cl._18_	0.18
400	[Mg_.77_Al_.23_(OH)_2_]Cl._23_	0.23
500	[Mg_.71_Al_.29_(OH)_2_]Cl._06_	0.29

### 3.2. Anion Exchange

The hydrotalcites in the chloride form were treated with various other anions. This was accomplished by dispersing the chloride form in 0.5 molar solutions containing nitrate, carbonate, sulfate, phosphate and acetate with subsequent centrifugation. This process was repeated three times. The final product was rinsed thoroughly with distilled water. 

### 3.3. TGA/EGA

The thermal decomposition analysis and EGA by mass spectrometry data were collected on a Hiden CatLAB micro-reactor (Hiden Analytical Inc., Livonia, MI, USA) combined with the Hiden QIC 20 quadrupole mass spectrometer (Hiden Analytical Inc., Livonia, MI, USA) with a temperature program heating rate of 10 degrees Celsius per minute from ambient to 650 °C in helium atmosphere. Additionally, thermal gravimetric analysis data were also collected on a TA Instrument Q600 combined with the Hiden HPR20 quadrapole mass spectrometer (Hiden Analytical Inc., Livonia, MI, USA) with the same heating rate as the CatLAB and inert argon atmosphere. The redundancy in measurements allow for cross-validation of the results.

### 3.4. X-ray Diffraction

The X-ray diffraction data were collected on a Bruker D-8 diffractometer (Broker AXS Inc., Madison, WI, USA) utilizing Cu Ka radiation with a step rate of 3 seconds per step and a step size of 0.02 degrees 2θ.

## 4. Conclusions

Based upon the thermal decomposition curves and mass spectra data, several conclusions can be drawn concerning the conversion of hydrotalcite into a mixed metal oxide. The first is that it is very important to conduct thermal degradation studies on samples that are essentially free of interfering anions, such as carbonate. Secondly, it is advantageous to conduct the synthesis of the hydrotalcite under hydrothermal conditions to promote crystal growth. This helps minimize the tendency to uptake carbonate and yields well-ordered material with high crystallinity and a large plate size. Thirdly, the observed dehydroxylation curves fit very nicely with a stepwise loss of water with the formation of intermediate oxyhydroxide species that correlate well with the level of aluminum substitution in the hydrotalcite. Lastly, the postulate that there are different thermal stabilities for the edge and center hydroxyl is not supported by the data of highly crystalline hydrotalcite, where edge hydroxides are a minor portion of the total.
